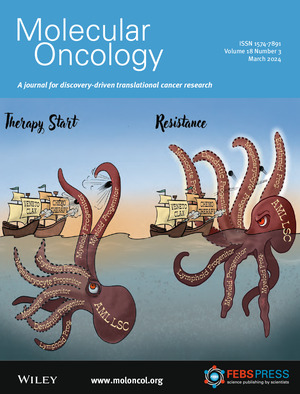# Issue Information

**DOI:** 10.1002/1878-0261.13457

**Published:** 2024-03-07

## Abstract

An Arms Race against Leukemia Resistance: Clinically relevant drugs predominantly target the myeloid progenitor lineage, while monocyte or stem cell‐like states can evade current AML treatments, akin to the lineage plasticity of leukemic stem cells (LSC). Read the Viewpoint article by Waclawiczek A., Leppä A.M., Renders S., Trumpp A. in pp. 475–478.

Illustration credit: Evelyn Strehlow.